# Heterologous prime-boost BCG with DNA vaccine expressing fusion antigens Rv2299c and Ag85A improves protective efficacy against *Mycobacterium tuberculosis* in mice

**DOI:** 10.3389/fmicb.2022.927031

**Published:** 2022-10-04

**Authors:** Juan Wu, Zhidong Hu, Shui-Hua Lu, Xiao-Yong Fan

**Affiliations:** ^1^Shanghai Public Health Clinical Center, Key Laboratory of Medical Molecular Virology of MOE/MOH, Fudan University, Shanghai, China; ^2^National Medical Center for Infectious Diseases of China Shenzhen Third People Hospital, Southern University of Science and Technology, Shenzhen, China

**Keywords:** heterologous prime–boost, Bacille Calmette–Guerin, DNA vaccine, Rv2299c, *Mycobacterium tuberculosis*

## Abstract

The development of heterologous prime-boost regimens utilizing Bacille Calmette–Guerin (BCG) as the priming vaccine is a promising approach to improve the efficacy of vaccination against tuberculosis (TB). In this study, we examined the ability of a DNA vaccine that expressed a fusion of antigens Rv2299c and Ag85A to boost BCG immunity and protection against *Mycobacterium tuberculosis* (*Mtb*) in Balb/c mice. The fusion DNA vaccine was moderately immunogenic and afforded some protection when used on its own. After a priming BCG vaccination, the DNA boost significantly amplified Th1-type cell-mediated immunity compared to that resulting from either BCG or DNA immunization. In the DNA-boosted mice, Ag-specific CD4^+^ and CD8^+^ T cells that were mono-positive for IFN-γ alone were the most prominently expanded in infected lungs. The protective efficacy afforded by BCG against challenge infection was greatly improved by the DNA boost; bacterial loads were significantly reduced in both spleen and lung and histological damage in the lung was less. The use of a DNA vaccine containing the fusion antigens Rv2299c and Ag85A to boost BCG may be a good choice for the rational design of an efficient vaccination strategy against TB.

## Introduction

Tuberculosis (TB), an infectious disease caused by *Mycobacterium tuberculosis* (*Mtb*), is associated with high morbidity and mortality and poses a major global public health problem. The emergence of HIV-associated TB and the increasing frequency of multi-drug resistant *Mtb* infections have created an emergency and added emphasis to the need to develop better control strategies. Currently, Bacille Calmette–Guerin (BCG), which was developed nearly 100 years ago, is the only available vaccine to prevent TB. BCG is the most widely administered vaccine around the world and can provide 60–80% protection against childhood and extra-pulmonary forms of TB (Fine, 1995; Trunz et al., 2006; Young et al., 2008; Mangtani et al., 2014; McShane, 2014). But the beneficial effect of the vaccine wanes over time, resulting in highly inconsistent and inadequate protection against pulmonary TB, the transmitted form of the disease, and BCG is often ineffective when given to adults (Trunz et al., 2006; Mangtani et al., 2014). Consequently, it has not had a major effect on the global burden of TB (Fine, 1995; Trunz et al., 2006; Young et al., 2008; Mangtani et al., 2014; McShane, 2014). Re-vaccination to boost the initial immune responses is an attractive strategy (Cervantes-Villagrana et al., 2013; Tan et al., 2014) but homologous boosting with BCG is of limited benefit (Nemes et al., 2018). Heterologous prime–boost strategies that rely on selected antigens that are present in BCG and *Mtb* may be an optimal approach.

DNA vaccines offer the advantage of endogenous antigen production within antigen-presenting cells, leading to stimulation of CD8^+^ in addition to CD4^+^ T cells (Li and Petrovsky, 2016; Suschak et al., 2017). They have proven successful in various animal models in treating and preventing cancer, autoimmunity, and infectious diseases (Abdulhaqq and Weiner, 2008; Ojha and Prajapati, 2021). We and others have described several DNA vaccines that can confer immune protection against *Mtb* (Fan et al., 2009; Wu et al., 2011; Kang et al., 2014; Xu et al., 2014; Ji et al., 2016; Hu et al., 2019). Consequently, DNA vaccines are especially promising candidates for clinical development; however, the efficacy of DNA vaccines expressing only one antigen of *Mtb* is limited (Kamath et al., 1999). The development of multigenic constructs expressing additional *Mtb* antigens might lead to better DNA vaccines against TB. Here we test the efficacy of a DNA vaccine expressing two *Mtb* antigens, Rv2299c and Ag85A, as a fusion protein.

The product of gene *Rv2299c*, Rv2299c, also denoted as HtpGMtb, is a member of the HSP90 family and has been reported as an effective inducer of dendritic cell maturation and enhancer of polyfunctional T cells and antimycobacterial action (Choi et al., 2017). Notably, the fusion of Rv2299c to ESAT6 or to Ag85B-ESAT6 enhanced immunoreactivity and boosted BCG protection against *Mtb* (Choi et al., 2017, 2020; Back et al., 2020). Therefore, Rv2299c is an excellent candidate for inclusion in the rational design of an effective multiantigen TB vaccine. Many studies devising new TB vaccines have naturally focused on strong T-cell-stimulating antigens, such as the antigen 85 complex (Ag85) and ESAT6 (early secretory antigenic target-6). However, ESAT6 is an immunodominant *Mtb* antigen that is not produced by BCG and accordingly has a key role in TB-detection assays, such as interferon-gamma (IFN-γ) release assays (IGRAs). The IGRAs containing ESAT6 are highly sensitive and specific to distinguish between BCG vaccination and *Mtb* infection. Therefore, the inclusion of ESAT6 in new vaccines may not be desirable. We selected Ag85A as a fusion partner with Rv2299c because it is among the major immunogenic *Mtb* secretory proteins with the ability to stimulate humoral and cell-mediated immune responses and protection in mice against *Mtb* challenge (Huygen et al., 1996). Thus, Ag85A was regarded as one of the promising antigens in anti-TB DNA vaccine design (Huygen et al., 1996). We previously demonstrated that BCG primed Ag85A DNA boosting reduced the bacterial load in mouse lungs and spleen after a challenge with H37Rv (Kang et al., 2014). Here, we directly compare the immunogenicity and protective efficacy of a DNA vaccine expressing a fusion of antigens Rv2299c and Ag85A (pVAX1Rv2299cAg85A) and DNA vaccines expressing these antigens separately (pVAX1Rv2299c, pVAX1Ag85A). We found that this fusion DNA vaccine was moderately immunogenic. It afforded some protection against challenge infection with *Mtb* in mice and boosted BCG-induced immunity and protection in association with an enhancement of production of multifunctional CD4^+^ T cells.

## Materials and methods

### Animals

Female Balb/c mice, 5–6 weeks of age, were obtained from Shanghai Public Health Clinical Center (SPHC). The mice were kept under pathogen-free conditions at SPHC. The mice were maintained in a room with controlled temperature (20–22°C), humidity (50–60%), and light (12 h:12 h) with free access to standard rodent chow and water. All experiments were performed under the guidelines of the Institutional Animal Care and Use Committee and the protocol was approved by the committee on the Ethics of Animal Experiments at the SPHC (Mouse Protocol Number: SYXK-2010-0098). Mice were killed by CO2 inhalation, and care was taken to minimize suffering.

### The preparation of recombinant protein and DNA vaccines

The procedures for cloning and expression of *Mtb rv3804c* and *rv2299c* for the preparation of Ag85A protein, Rv2299c protein, and DNA were as follows.

To produce the separate recombinant Ag85A and Rv2299c proteins, the corresponding gene was amplified by PCR using *Mtb* H37Rv ATCC 27294 genomic DNA as a template. The PCR product was digested with *BamHI* and *EcoRI* and inserted into a pET28a(+) plasmid (Novagen, Madison, WI). Plasmids containing recombinant pET28a(+)Rv3804c or pET28a(+)Rv2299c were transformed into *E. coli BL21*(DE3) cells with heat shock for 1 min at 42°C. After cell disruption by ultra-sonication, soluble protein Ag85A or Rv2299c was purified using Ni-NTA resin as previously described with slight modifications (Wu et al., 2021). To remove endotoxins, the dialyzed recombinant protein was incubated with High-Performance Endotoxin Removal Agarose Resin (Yeasen, 20518ES10). Finally, the purified endotoxin-free recombinant protein was filter-sterilized and frozen at −80°C. The protein concentration was estimated using a Detergent Compatible Bradford Protein Assay Kit (Beyotime, P0006C) with bovine serum albumin as a standard.

A 2838 bp *rv2299c-rv3804c* fusion gene was constructed. First, a 1944 bp fragment of *rv2299c* was amplified from the *Mtb* H37Rv genome by PCR using a forward primer containing a *BamHI* restriction enzyme site: (5′- CG*GGATCC*ATGAACGCCCATGTCGAG-3′), and the reverse primer containing an *EcoRI* restriction enzyme site (5’-CG*GAATTC*CAAGGTACGCGCGAGACG-3′). The resulting PCR product was purified and ligated with *BamHI/EcoRI*-digested pVAX1 vector (Novagen, Madison, WI) using T4 DNA ligase (NEB). The ligated product was transformed into *E. coli* Top10 strain and named pVAX1Rv2299c. The nucleotide sequence encoding the Rv2299c was analyzed by GENEWIZ Ltd. Suzhou, China and compared by BLAST analysis against the whole genome of *Mtb* as registered in the GenBank database. An 888 bp sequence containing *rv3804c* was amplified from the *Mtb* H37Rv genome by PCR using a forward primer containing an *EcoRI* restriction enzyme site: (5’-CG*GAATTC*ATGTTTTCCCGGCCGGG-3′), and a reverse primer containing an *XbaI* restriction enzyme site (5′- GC*TCTAGA*GGCGCCCTGGGGC-3′; GENEWIZ Ltd. Suzhou, China). The resulting PCR product was purified and ligated with *EcoRI/XbaI-*digested pVAX1Rv2299c or with pVAX1 using T4 DNA ligase (NEB). The ligated product was transformed into *E. coli* Top10. The resulting recombinant plasmids were named pVAX1Rv2299cAg85A and pVAX1Ag85A. The nucleotide sequences encoding the protein were analyzed by GENEWIZ Ltd. Suzhou, China, and compared by BLAST analysis against the whole genome of *Mtb* in the GenBank database.

### Vaccination

For DNA vaccination, three doses of 100 μg DNA vaccine were injected intramuscularly (i.m.) into each hind leg in a volume of 100 μl at 2-week intervals. The BCG Danish strain was injected subcutaneously (s.c.) with 5 × 10^6^ colony-forming units (CFU) in 200 μl PBS around the hind legs. PBS was used as a negative control. For the evaluation of primary T-cell immune responses, at 6 weeks after the primary immunization, the mice were sacrificed and the lungs and spleens were aseptically removed for assays of antigen-specific cellular immune responses. For the evaluation of recall immune responses after infection, the mice were challenged by the introduction of the *Mtb* virulent strain H37Rv into the lungs 6 weeks after the primary immunization and maintained in an ABSL-3 animal facility. Four weeks later, the animals were killed, and the lungs were sampled.

### Lymphocyte isolation

For the separation of single lung cells, the lungs were sterilely removed and minced with scissors. Tissue pieces were digested for 30 min at 37°C with 10 U of DNase I (Thermo) and 1 mg/ml of collagenase IV (Invitrogen) in 10% FBS RPMI1640 medium then the digest was passed through a 70-μm cell strainer (Fisher Scientific) by gently squashing with a syringe plunger. Red blood cells were lysed with lysis buffer. For the separation of single splenocytes, the spleens were mechanically disrupted, single cells were filtered through mesh gauze and red blood cells were lysed. The lung lymphocytes and splenocytes were washed twice and re-suspended in a 10% FBS RPMI1640 medium.

### ELISPOT assay

Interferon (IFN)-γ ELISPOT assay was performed according to the kit instructions (BD Biosciences). Briefly, 96-well plates were coated with capture antibody at 4°C overnight. The plates were washed and blocked with 10% FBS DMEM medium for 2 h at room temperature. The isolated single lung cells or splenocytes were added at 2 × 10^5^ cells/well or 4 × 10^5^ cells/well together with Ag85A (10 μg/ml) or PPD (10 μg/ml) as specific antigenic stimuli. Positive control wells contained PMA (50 ng/ml, Sigma) plus ionomycin (1 μg/ml, Sigma), and cells incubated with medium only (10% FBS RPMI1640) were used as negative controls. The cells were stimulated in a 5% CO2 and humidified incubator at 37°C for 20 h. The stimulated cells were washed with deionized water and PBST (PBS containing 0.05% Tween-20) and then incubated with the detection antibody for 2 h. After washing again with PBST, the plates were incubated with streptavidin-HRP for an additional 1 h. After washing with PBST and PBS, the AEC substrate solution was added and incubated for 30 min before rinsing with water. The plates were air-dried and analyzed with an Immunospot Reader (Champspot III, Beijing Sage Creation Science).

### Intracellular staining and flow cytometry analysis

The isolated single lung cells or splenocytes were stimulated with Ag85A (10 μg/ml), Rv2299c (10 μg/ml), PPD (10 μg/ml) or Rv2299c-Ag85A (10 μg/ml) in 96-well plates in a volume of 200 μl at 37°C for 10 h, then GolgiPlug and GolgiStop were added and the cells were incubated for an additional 5 h (BD Biosciences, San Jose, CA, United States). PMA (50 ng/ml) plus ionomycin (1 μg/ml) stimulation was used as a positive control and cells incubated with 10% FBS RPMI1640 were used as a negative control. After stimulation, the cells were washed with PBS containing 2% fetal bovine serum and then incubated with a mixture of surface antibodies for 30 min at 4°C. After washing, the cells were fixed and rendered permeable with fix/perm buffer for 30 min at 4°C (BD Bioscience). After washing, the cells were incubated with antibodies against intracellular cytokines for another 40 min at 4°C. Then the cells were resuspended in PBS and acquired on a FACS Fortessa (Becton Dickinson, Franklin Lakes, New Jersey, United States). Results were analyzed by using FlowJo software (Tree Star, Ashland, OR, United States), and the doublet events were excluded by FSC-A/FSC-H gating.

### Antibodies

The antibodies used in this study were: CD3-eFlour450(17A2), CD4-APC-eFlour780(GK1.5),CD8-PerCP-Cy5.5(53–6.7),CD44-FITC(IM7),CD62L-Brilliant Violet650™(MEL-14), IL-2-PE(JES6-5H4), IFN-γ-APC(XMG1.2), TNF-a-PE-Cy7 (MP6-XT22) and IL-17A-Alexa Flour®700(TC11-18H10.1). All were from eBioscience™ and BioLegend®.

### Mycobacterial challenge and bacterial counting

At 6 weeks after the primary immunization, mice were subjected to bacterial challenge in groups of five in a BSL-3 animal facility. Mice were exposed to an aerosol of *Mtb* H37Rv strain in an inhalation exposure system (GLAS-COL Model #A4212 099c, Terre Haute, IN, United States) to deposit a dose of 100 bacterial colony-forming units (CFU) per mouse as described (Hu et al., 2017). Four weeks after the challenge, mice were sacrificed and lung lobes were separated for enumeration of CFU, H&E staining, and immunological analysis. The right postcaval lobes were used for H&E staining, the right superior lobes were used for immunological analysis. The remaining lung lobes were used for CFU plating. Bacterial loads in the spleens were also determined by CFU plating.

### Histopathological analysis

The right superior lobes of the infected lung were fixed in formalin and embedded in paraffin. Embedded lung lobes were sectioned in a thickness of 5 mm, stained with H&E, and photographed using a Nikon Eclipse Ci-L microscope (Japan) fitted with a Hungary CaseViewer2.4 camera connected to a computer. The Image-Pro Plus program (Media Cybernetics) was utilized to objectively assess the level of inflammation present in each image. The inflammatory areas stained a more intense purple than the non-inflammatory areas. The percentage of the lung with granulomatous infiltration/consolidation, as so defined, was quantified by averaging from three to five lung sections from each mouse.

### Statistical analysis

Results were expressed as the mean ± standard error of the mean (SEM). Data sets were analyzed using two-tailed *p*-value and unpaired *t*-test or one-way or two-way ANOVA using GraphPad Prism 8. *p*-values smaller than 0.05 were considered to be statistically significant.

## Results

### Immunogenicity and protective efficacy in DNA vaccines expressing antigens Rv2299c and Ag85A singly or together

**Figure 1:**
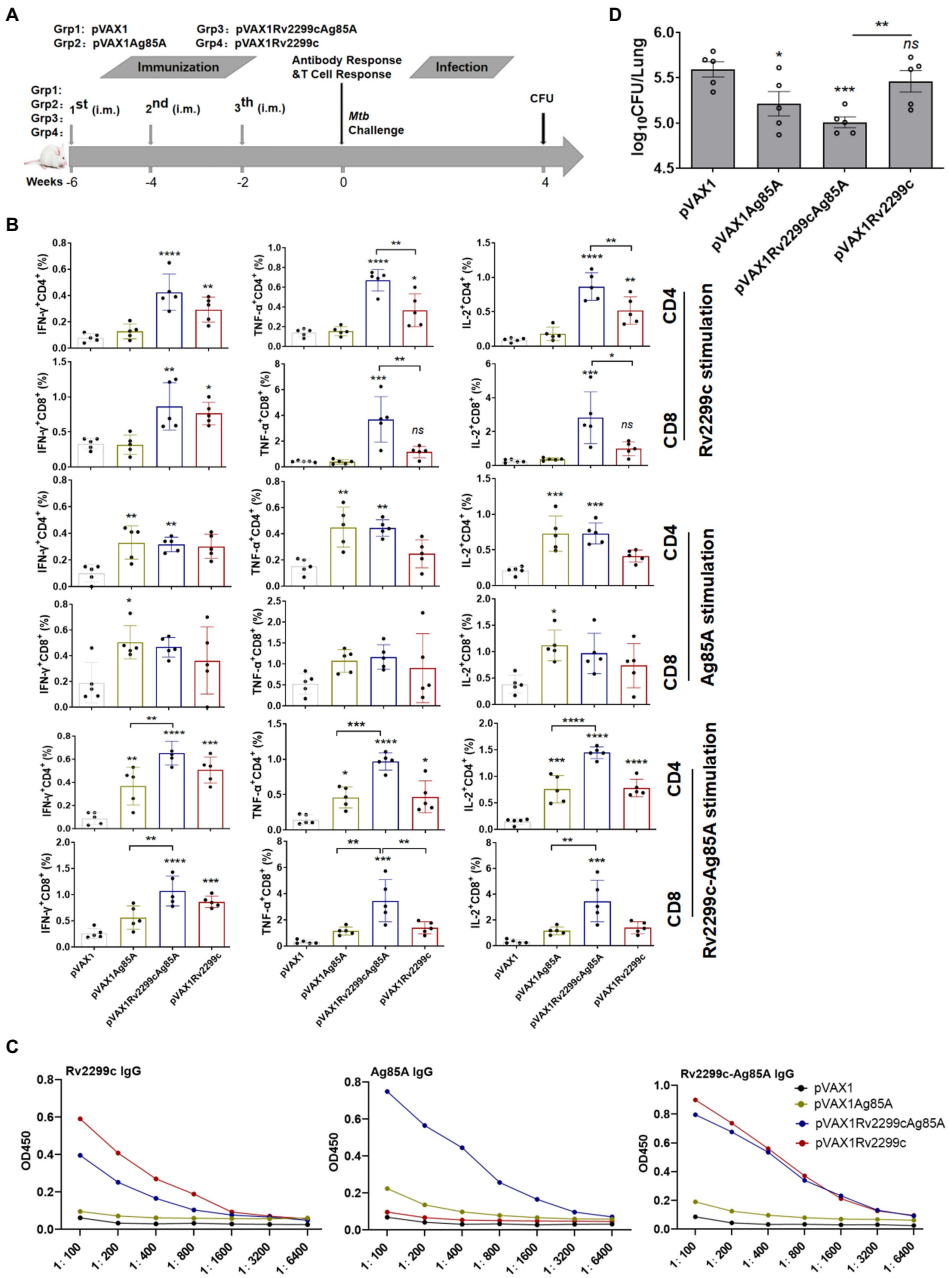
Preliminary demonstration that DNA vaccines expressing fusion antigens Rv2299c and Ag85A are moderately immunogenic and can afford protection. **(A)** Vaccination and infection schedule. Six weeks after the primary immunization, the mice were sacrificed, then splenocytes were collected and treated with Rv2299c (10 μg/ml) or Ag85A (10 μg/ml) at 37°C for 15 h in the presence of GolgiStop and intracellular cytokine staining was evaluated by flow cytometry. **(B)** The overall percentages of CD4^+^ or CD8^+^ T cells producing IFN-γ, TNF-α, or IL-2 after stimulation with Rv2299c or Ag85A or 2299c-Ag85A (*n* = 3 mice, one-way ANOVA, mean ± SEM). **(C)** Specific IgG responses to antigen Rv2299c or Ag85A or Rv2299c-Ag85A in serum assayed six weeks after the primary immunization (*n* = 3 mice). **(D)** Pulmonary bacterial burden, Log_10_ CFU, at four weeks after *Mtb* H37Rv challenge. (*n* = 5 mice, two tailed P and unpaired *t* test).Data are representative of two independent experiments with at least three mice per group.*p < 0.05, **p < 0.01,***p < 0.001, ****p < 0.0001: a significant difference of treatment groups from the appropriate controls (pVAX1 group). *ns*, no significant difference.

293 T cells transfected with DNA encoding the fused Rv2299c/Ag85A antigens (pVAX1Rv2299cAg85A) generated the expected 110 KDa molecular weight protein product (Supplementary Figure S1). In addition, the endotoxin content was measured by a LAL assay and its concentration was below 10 UE/mg in Rv2299c, Ag85A and Rv2299c-Ag85A after the Polymyxin treatment protein (Supplementary Figure S2). Moreover, the Rv2299c protein significantly increased BMDMs secretion of TNF-a,IL-6 and IL-1ß in a dose-dependent manner, and the concentration of 10 μg/ml stimulated the highest cytokines (Supplementary Figure S2).The DNA vaccines pVAX1Ag85A, pVAX1Rv2299c, and pVAX1Rv2299cAg85A were compared in Balb/c mice for immunogenicity and protection against challenge infection 6 weeks after the primary immunization (Figure 1A). Three vaccinations with pVAX1Rv2299c induced a robust response in Rv2299c-specific CD4^+^ Th1 T cells expressing IFN-γ, TNF-α, and IL-2 and in Rv2299c-specific CD8^+^ T cells that produced IFN-γ (Figure 1B). The TNF-α and IL-2 responses to Rv2299c were significantly greater if the vaccination was done with the DNA expressing the fusion antigen (pVAX1Rv2299cAg85A). Vaccination with pVAX1Ag85A induced robust responses in Ag85A-specific CD4^+^ and CD8^+^ T cells expressing IFN-γ, TNF-α, and IL-2, but the responses to Ag85A after pVAX1Rv2299cAg85A vaccination were not significantly greater (Figure 1B). Vaccination with pVAX1Rv2299cAg85A induced robust responses in Rv2299c-Ag85A-specific CD4^+^ and CD8^+^ T cells expressing IFN-γ, TNF-α, and IL-2, and the responses to Rv2299c-Ag85A after pVAX1Rv2299cAg85A vaccination were significantly greater (Figure 1B). A gating strategy to identify multifunctional T cells was established (Supplementary Figure S3) and used to define differences in the frequency of cells producing one, two, or three of the cytokines (Supplementary Figure S4). This subset analysis of Rv2299c-specific multifunctional T cells showed that after vaccination with either pVAX1Rv2299cAg85A or pVAX1Rv2299c the proportion of CD4^+^ T cells that were triple-cytokine-positive IFN-γ^+^TNF-α^+^IL-2^+^, dual-positive TNF-α^+^IL-2^+^, or mono-positive IL-2^+^ were significantly expanded. Among the CD8^+^ T cells, the dual-positive TNF-α^+^IL-2^+^, mono-positive TNF-α^+^, and IL-2^+^ subsets were increased significantly after pVAX1Rv2299cAg85A but not after pVAX1Rv2299c vaccination (Supplementary Figure S4). Subset analysis of the Ag85A-specific CD4^+^ T cells showed that the triple-cytokine-positive IFN-γ^+^TNF-α^+^IL-2^+^, the dual-positive TNF-α^+^IL-2^+^, and mono-positive IL-2^+^ subsets were significantly expanded after pVAX1Rv2299cAg85A and pVAX1Ag85A, and there was no significant difference between the vaccines; Ag85A-specific CD8^+^ T-cell subset responses were small and not significant (Supplementary Figure S4). Specific IgG antibody responses to the vaccines are shown in Figure 1C. The Rv2299c or Rv2299c-Ag85A-specific IgG response from pVAX1Rv2299c vaccination was similar to that from pVAX1Rv2299cAg85A vaccination. However, the Ag85A-specific response to pVAX1Rv2299cAg85A gave a 4-fold higher titer than the response to pVAX1Ag85A (Figure 1C). In a simple, rapid test of protective efficacy (Figure 1D), vaccination with pVAX1Ag85A moderately reduced the bacterial burden in the lungs 4 weeks after challenge infection, and although vaccination with pVAX1Rv2299c had minimal effect, the fusion vaccine had the greatest effect.

### Boosting with DNA expanded of the memory T-cell population primed by BCG vaccination

To confirm DNA vaccine enhancing BCG-primed antibody responses, serum was analyzed by ELISAs. Specific IgG antibody responses to the vaccines are shown in Supplementary Figure S4. The Rv2299c or PPD-specific IgG response to BCG-DNA gave a 2-fold higher titer than the response to BCG. However, the Ag85A or Rv2299c-Ag85A-specific response to BCG-DNA gave a 5-fold higher titer than the response to BCG (Supplementary Figure S5). Moreover, we also investigated whether BCG-primed DNA vaccine results in the expansion of the memory T-cell population, we analyzed the expression change of CD62L and CD44 on CD4^+^ and CD8^+^ T cells using flow cytometry. As shown in Supplementary Figure S6, BCG-primed DNA induced the expansion of effector/memory T cells by significantly down regulating CD62L and up regulating CD44 in CD4^+^ and CD8^+^ T-cells when compared with BCG or DNA vaccine. Moreover, BCG-primed DNA induced the expansion of central/memory T-cells by significantly up regulating CD62L and up regulating CD44 in CD4^+^ and CD8^+^ T-cells when compared with DNA vaccine, but only expanded central/memory T-cells CD8^+^ T-cells when compared with BCG vaccine.

### Boosting with DNA increased the IFN-γ responses primed by BCG vaccination

**Figure 2:**
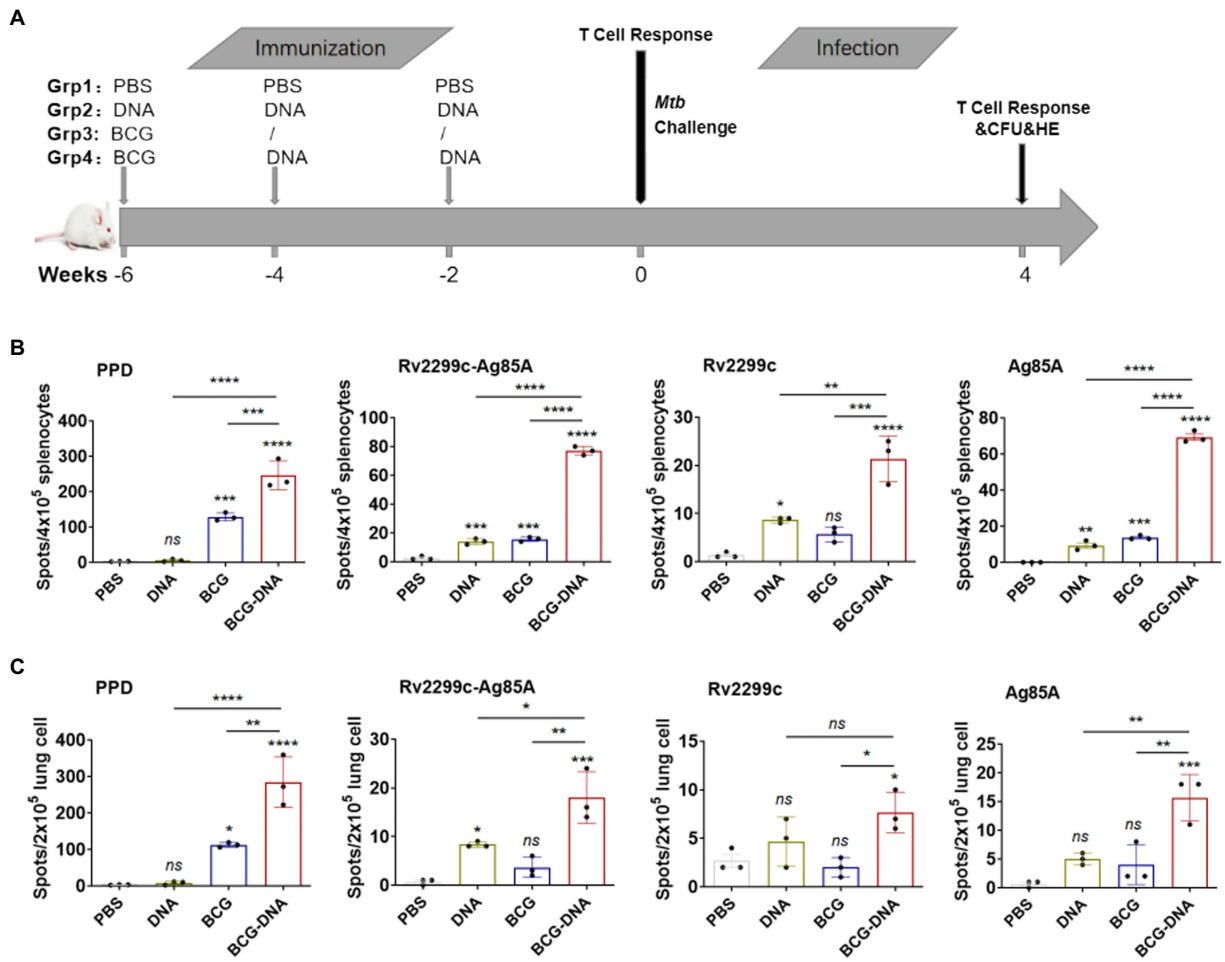
ELISPOT assay of antigen-specific IFN-γ-production by T cells from immunized mice prior to infection. **(A)** Vaccination and infection schedule. At 6 weeks after the primary immunization, cells from spleens **(B)** or lungs **(C)** of immunized mice (*n* = 3) were isolated and incubated with PPD, Rv2299c, Ag85A or Rv2299c-Ag85A protein for ex vivo IFN-γ ELISPOT assays. Representative responses revealed by Elispot assays and quantified are shown as spot-forming units (*n* = 3 mice, one-way ANOVA, mean ± SEM). *p < 0.05, **p < 0.01, ***p < 0.001, ****p < 0.0001: a significant difference of treatment groups from the appropriate controls (PBS group). *ns*, no significant difference.

Based on this finding that the fusion DNA vaccine was moderately immunogenic and afforded some protection in a mouse model, we tested it as a booster for BCG immunization (Figure 2A). Spleen and lung cells that were isolated immediately after the final immunization were analyzed by ex vivo ELISPOT assay for IFN-γ-production in response to PPD (10 μg/ml), Rv2299c-Ag85A (10 μg/ml), Rv2299c (10 μg/ml) or Ag85A (10 μg/ml) stimulation. Typical IFN-γ ELISPOT results from spleens and lungs are shown and quantified in Figures 2B,C respectively. As expected, BCG vaccination induced PPD-specific immune responses, and the responses were significantly greater in DNA-boosted mice. Although the Rv2299c-Ag85A or Rv2299c or Ag85A-specific cell responses induced by DNA or BCG were small, mice receiving DNA boosts secreted higher levels of IFN-γ compared with DNA or BCG immunization alone. Flow cytometry indicated that boosting increased the percentages of CD8^+^ T cells producing IFN-γ, TNF-α, and IL-17A in response to PPD in the lung, and IFN-γ, TNF-α, and IL-2 in the spleen but the differences did not reach statistical significance (Supplementary Figure S7).

### Boosting with DNA increased poly-functional T-cell responses primed by BCG vaccination

**Figure 3:**
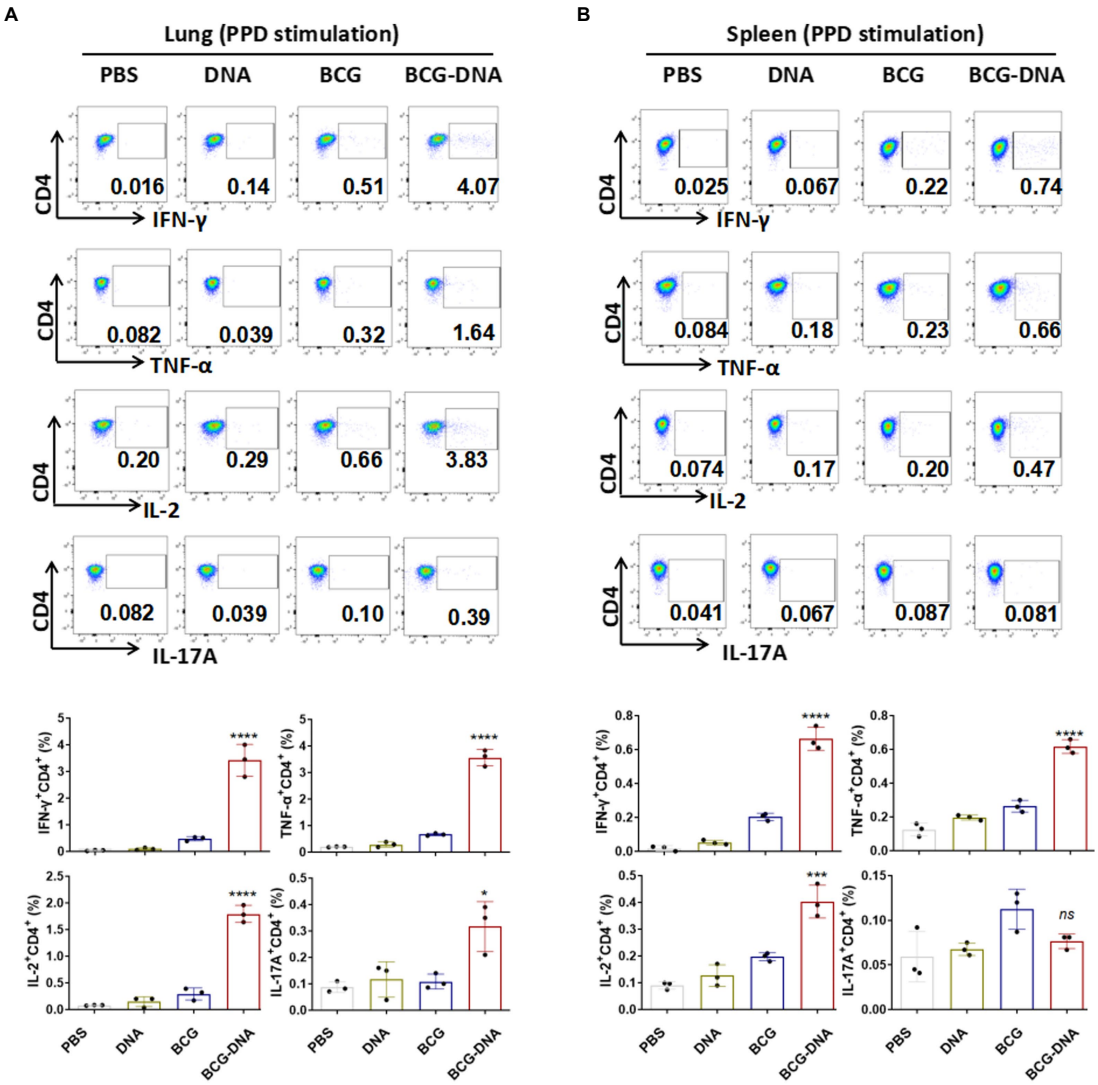
Flow cytometric analysis of cytokine production in T cells from immunized mice prior to infection. Six weeks after the primary immunization, the mice were sacrificed, cells were harvested from the mice (*n* = 3) and treated with PPD (10 μg/ml) at 37°C for 15 h in the presence of GolgiStop. Production of IFN-γ, TNF-α, IL-2 and IL-17A in the lung cells **(A)** and splenocytes **(B)** were determined by flow cytometry. Representative flow cytometric plots of IFN-γ, TNF-α, IL-2 and IL-17A staining in CD4^+^ T cells (top) and the proportions of CD4^+^ T cells producing cytokines (bottom) are shown. (*n* = 3 mice, one-way ANOVA, mean ± SEM). *p < 0.05, ***p < 0.001, ****p < 0.0001: a significant difference of treatment groups from the appropriate controls (PBS group). *ns*, no significant difference.

**Figure 4:**
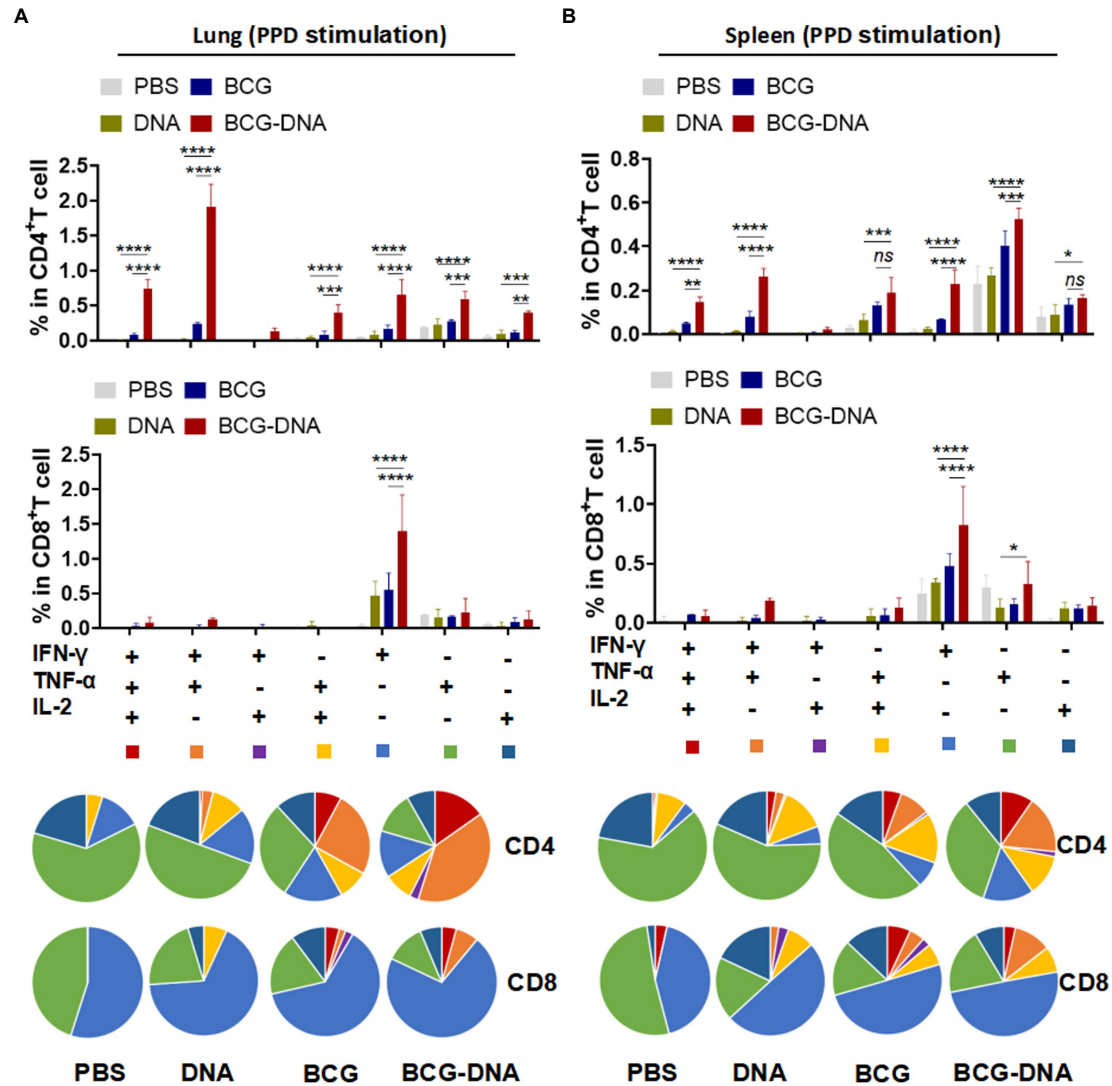
Induction of Ag-specific polyfunctional T-cells in lungs and spleens of immunized mice. Six weeks after the primary immunization, the mice were sacrificed, and their lung and spleen cells were collected and treated with PPD (10 μg/ml) at 37°C for 15 h in the presence of GolgiStop. Ag-specific T cells secreting IFN-γ, TNF-α and IL-2 were distinguished by flow cytometry and classed into seven sub-populations based on the production of single or multiple cytokines. The percentages of the seven sub-populations as components of the total CD4^+^ or CD8^+^ T cells in lung **(A)** and spleen **(B)** and the pie chart analysis are shown. (*n* = 3 mice, two-way ANOVA, mean ± SEM). *p < 0.05, **p < 0.01, ***p < 0.001, ****p < 0.0001: a significant difference of treatment groups from the appropriate controls (PBS group). *ns*, no significant difference.

At 6 weeks after the primary immunization, lung cells and splenocytes were harvested and stimulated with PPD (10 μg/ml) or Rv2299c-Ag85A (10 μg/ml) for 15 h in the presence of GolgiStop then analyzed by flow cytometric ICS detection of Ag-specific CD4^+^ T cells producing IFN-γ, TNF-α, IL-2, and IL-17A. Figure 3 shows representative dot plots and frequencies of CD4^+^ T cells responding to PPD. The proportions producing IFN-γ, TNF-α, and IL-2 were significantly expanded both in the lung cells (Figure 3A, bottom) and splenocytes (Figure 3B, bottom), but the PPD-specific CD4^+^ T cells producing IL-17A were significantly boosted only in the lung (Figure 3A). The PPD responses of CD8^+^ T cells from spleens and lungs were similarly boosted by the DNA vaccine but only the IFN-γ enhancement reached statistical significance (Supplementary Figure S7). The polyfunctional responses of the CD4^+^ and CD8^+^ T cells to PPD are shown in Figure 4. Among CD4^+^ T cells from the lung, significant expansion was seen in the triple-cytokine-positive IFN-γ^+^TNF-α^+^IL-2^+^, the dual-positive IFN-γ^+^TNF-α^+^, TNF-α ^+^IL-2^+^, and mono-positive IFN-γ, TNF-α ^+^, and IL-2^+^ subsets were significantly expanded (Figure 4A), whereas, in the CD8^+^ T cells, only the mono-positive IFN-γ^+^ subset was expanded (Figure 4A). In the spleen, in PPD-specific CD4^+^ T cells, the triple-cytokine-positive IFN-γ^+^TNF-α^+^IL-2^+^, the dual-positive IFN-γ^+^TNF-α^+^, and mono-positive IFN-γ^+^ and TNF-α^+^ subsets were significantly expanded, whereas, in the CD8^+^ T cells, only the mono-positive IFN-γ^+^ subset was boosted (Figure 4B). However, the Rv2299c**-**Ag85A responses of only CD4^+^ splenocytes producing TNF-α with IL-2 were significantly expanded by the DNA vaccine (Supplementary Figure S8). The polyfunctional responses of the CD4^+^ and CD8^+^ cells to Rv2299c**-**Ag85A are shown in Supplementary Figure S9. Only CD4^+^ T cells from the spleens, the dual-positive TNF-α ^+^IL-2^+^, mono-positive TNF-α ^+^, and IL-2^+^ subsets were significantly expanded. Taken together, these data demonstrated that DNA boost induced higher levels of poly-functional antigen-specific CD4^+^ and CD8^+^ T-cell responses primed by the BCG vaccine.

### Protective efficacy against challenge with *Mtb* in BCG-primed DNA-boosted mice

**Figure 5:**
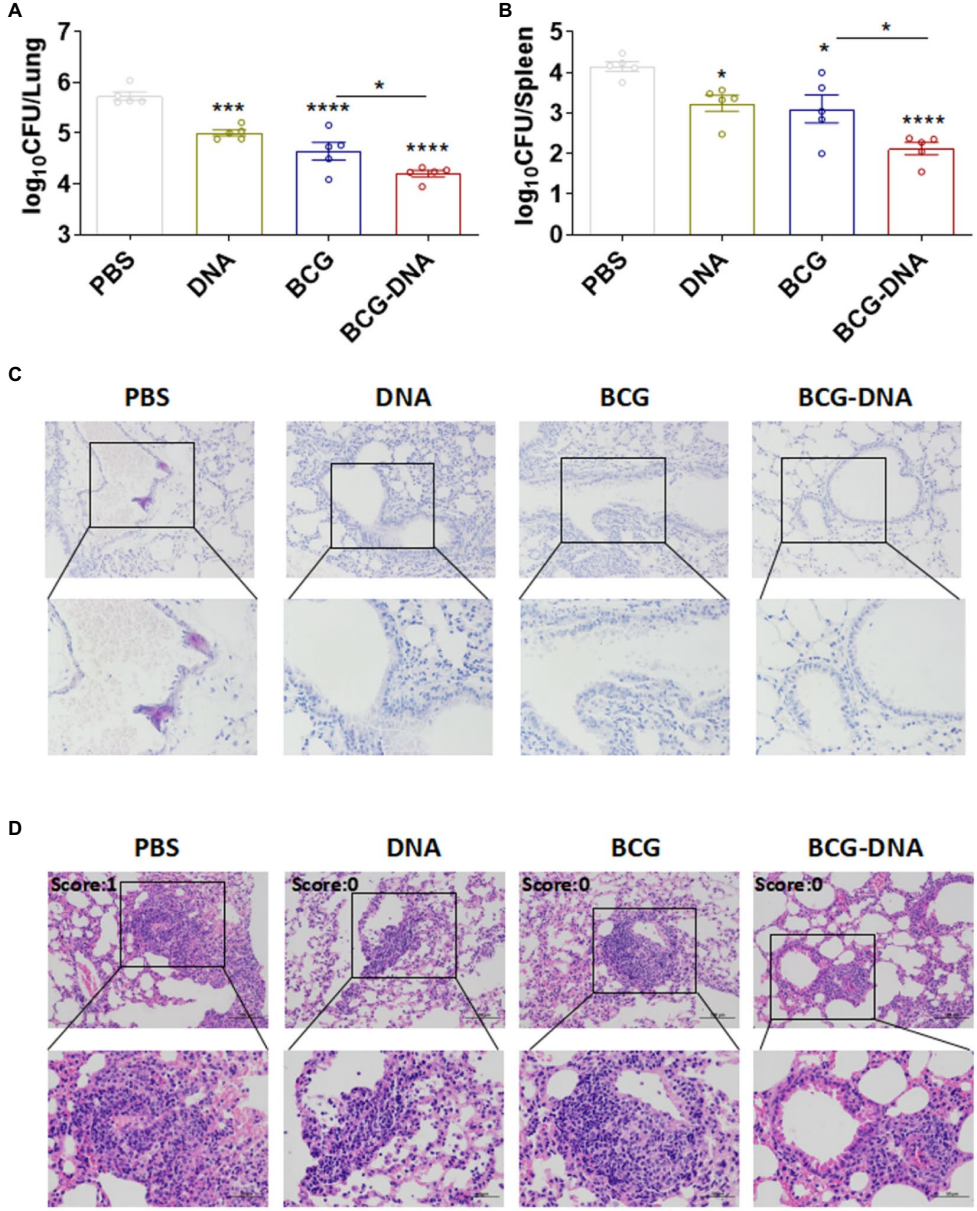
Enhanced protection by the BCG prime-DNA boost regimen. Six weeks after the primary immunization, mice (*n* = 5) were aerosol-challenged with *Mtb* H37Rv then sacrificed at four weeks post-infection. Bacterial loads in the lungs **(A)** and spleens **(B)** are shown as the mean log_10_ CFU/organ ± SEM. **(C)** The histopathology of lung tissues assessed in sections stained by acid-fast staining. Scale bars; 100 μm (top: original magnification ×200) and 50 μm (bottom: original magnification ×400). **(D)** H&E staining of the lungs of infected mice at four weeks post-infection (*n* = 5 mice, one-way ANOVA, mean ± SEM). *p < 0.05, **p < 0.01, ***p < 0.001, ****p < 0.0001: a significant difference of treatment groups from the appropriate controls (PBS group). *ns*, no significant difference.

To directly assess the protective efficacy of these prime-boost regimens, mice were challenged with *Mtb*, and the bacterial burden and histopathology were evaluated 4 weeks post-infection (Figure 5). In DNA-boosted mice, lung inflammation, and lesion size were significantly ameliorated when compared to either the BCG-vaccinated mice or DNA-vaccinated mice (Figures 5C,D). Moreover, bacterial burdens in the lungs and spleens were significantly lower in DNA-boosted mice than in BCG-vaccinated mice or DNA-vaccinated mice (Figures 5A,B). Thus, DNA vaccination boosted the protection induced by BCG.

### Multifunctional T-cells after challenge with *Mtb* in BCG primed DNA boosted mice

**Figure 6:**
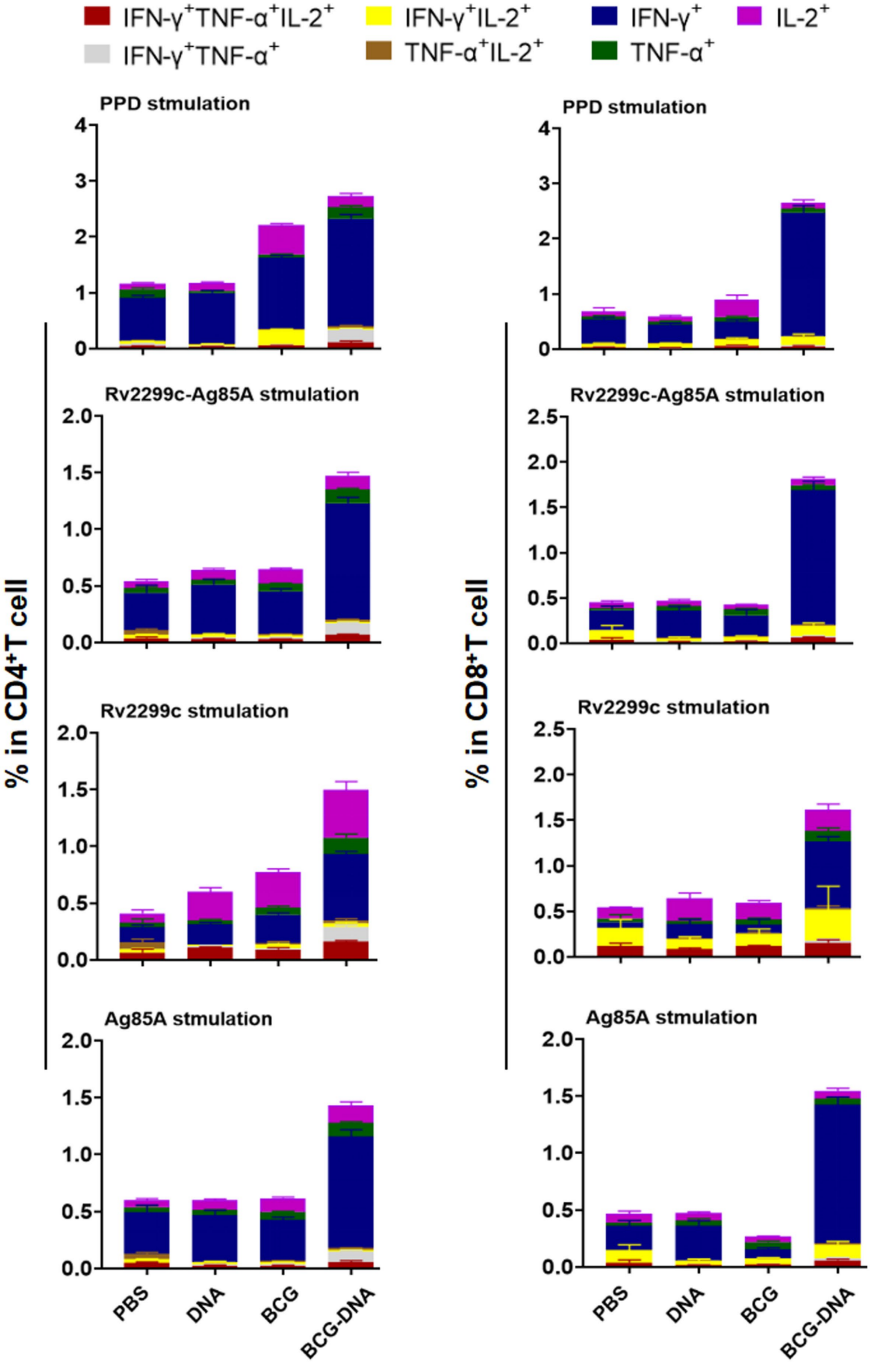
Polyfunctional CD4^+^ and CD8^+^ T-cell populations in the lungs of immunized mice after H37Rv challenge. Four weeks after infection of immunized mice pulmonary lymphocytes were collected and stimulated ex vivo with PPD, Ag85A or Rv2299c or Rv2299c-Ag85A. Frequencies of antigen-specific CD4^+^ (left) and CD8^+^ (right) T cells producing single or multiple cytokines were analyzed by flow cytometry. (*n* = 5 mice, mean ± SEM).

Four weeks after the H37Rv challenge, lung cells were harvested and stimulated with PPD, Ag85A, or Rv2299c for 15 h in the presence of GolgiStop and analyzed by cytometric ICS detection of Ag-specific, multifuctional T cells producing IFN-γ, TNF-α, and IL-2. Lung CD4^+^ and CD8^+^ T cells from challenge-infected mice that were stimulated with PPD, Rv2299c-Ag85A, Ag85A, or Rv2299c contained a majority population that was mono-positive IFN-γ alone and this was expanded to the most in CD4^+^ and CD8^+^ T cells from BCG-DNA-vaccinated mice (Figure 6). This expansion was most striking in the CD8^+^ T cells, increasing 7-fold in response to PPD stimulation, 9-fold in response to Rv2299c-Ag85A, 15-fold in response to Ag85A, and 8-fold in response to Rv2299c compared to cells from the BCG-vaccinated infected lungs.

## Discussion

Although DNA vaccines are traditionally considered to be the priming vector in the design of vaccine strategies, DNA-based anti-TB vaccines are often used to boost BCG since the majority of the human population is already BCG-vaccinated. Numerous studies in mice have shown that regimens with BCG prime and DNA vaccination boost enhance protective efficacy against *Mtb* infection compared to BCG vaccination alone (Derrick et al., 2004; Rouanet and Locht, 2010; Dey et al., 2011; Kang et al., 2014; Xu et al., 2014). In this study, we confirmed that immunization with DNA on its own could protect mice against subsequent *Mtb* challenge infection to a modest degree and that DNA boosting significantly enhanced the immune protection in BCG-primed mice (Figures 1, 5). Moreover, BCG-DNA immunization induced CFUs reduction in the lung 4 weeks after challenge compared with BCG vaccination. The CFU data from the lung indicate the level of circulating T cells capable of entering inflammatory sites in the tissues (Orme, 2006); hence, BCG-DNA might recruit more circulating T cells to the site of inflammation in the lung than BCG. Likewise, in the spleen, BCG-DNA yielded significantly fewer CFUs than BCG 4 weeks after challenge, indicating that in the BCG-DNA-immunized groups, higher levels of sensitized T cells were generated in the spleen. Higher numbers of these cells also entered the circulation via the thoracic duct from lymph nodes draining the sites of vaccine inoculation (Orme, 2006). Therefore, the T cells that are recruited and sensitized by BCG-DNA vaccination would be able to inhibit or delay *Mtb* replication and activation in the lung and consequently improve the protective effect of BCG against *Mtb*.

Prime-boost immunization can significantly augment the breadth of the induced immune responses, possibly because the divergent cell-targeting and antigen-processing routes complement one another and because the diversity of epitopes is greater than with either agent alone (Wu et al., 2005). In our study, the fusion of Rv2299c to Ag85A resulted in a vaccine that gave greater T-cell cytokine responses to Rv2299c than was obtained by vaccinating with Rv2299c alone. Moreover, the fusion vaccine gave responses to Rv2299c-Ag85A that were greater than the responses obtained after vaccination with Ag85A alone. In contrast, the fusion vaccine gave responses to Ag85A that were not greater than the responses obtained after vaccination with Ag85A alone. The basis of this difference remains to be explored but may contribute to the protective superiority of the fusion compared to the separate antigens shown in Figure 1. The fusion vaccine elicited typical protective cellular immune responses. Before the challenge infection, vaccination with the fusion DNA, BCG, or BCG-DNA-boost all increased the proportion of T cells that were multifunctional (Figures 4; Supplementary Figure S9). The expansion of the multifunctional populations was most prominent among CD4^+^ T cells from BCG-DNA-boosted mice. Among CD8^+^ T cells, mono-producers of IFN-γ predominated. The development of the challenge *Mtb* infection for 4 weeks markedly changed the cell profiles. The mono-producers of IFN-γ were expanded to predominate in the BCG-DNA-boosted mice (Figure 6). The BCG-DNA-boosted mice sustained the largest proportion of multifunctional CD4^+^ T cells during infection.

The potential limitations in our study were as follows including: Firstly, although the *in vitro* experiment and the immunogenicity experiments were repeated at least two times, the H37Rv infection experiment were only performed once with five mice per group, due to the lack of P3 lab resources during the COVID-19 pandemic. Secondly, this BCG-DNA regimen still needs to be optimized: changing the doses and inoculation times of DNA vaccination, and/or the time intervals between inoculations, might result in different levels of immune responses and different degrees of protection upon challenge.

Taken overall, these findings were consistent with the major roles of IFN-γ and multifunctional T cells in the protective immune response as previously described (Cooper et al., 1993; Aagaard et al., 2009; Abel et al., 2010; Scriba et al., 2010; Sweeney et al., 2011; Green et al., 2013; Hu et al., 2019; Waeckerle-Men et al., 2022). We conclude that the association of Rv2299c with Ag85A in the DNA fusion vaccine provided an attractive starting point for the development of a multi-antigen DNA vaccine to boost BCG immunity without impairing the use of the current antigen-specific TB diagnostic tests.

## Conclusion

In this study, we provide evidence that the fusion DNA vaccine was moderately immunogenic and afforded some protection when used on its own. After a priming BCG vaccination, the DNA boost significantly amplified Th1-type cell-mediated immunity, compared to that from either BCG or DNA immunization alone, and greatly improved the protective efficacy afforded by BCG against challenge infection with *Mtb*. Bacterial loads were significantly reduced in both spleen and lung and histological damage in the lung was less. Moreover, the BCG-DNA immunization regimen induced higher levels of recall T-cell responses after *Mtb* infection. These results indicate that the use of a DNA vaccine containing the fusion antigens Rv2299c and Ag85A to boost BCG is a good choice for the rational design of an efficient vaccination strategy against TB.

## Data availability statement

The original contributions presented in the study are included in the article/Supplementary material, further inquiries can be directed to the corresponding author.

## Ethics statement

The animal study was reviewed and approved by Shanghai Public Health Clinical Center.

## Author contributions

X-YF and JW conceived the project. JW carried out the experiments. JW, ZH, and X-YF conducted the analyses. JW and X-YF wrote the paper. X-YF and S-HL provided overall project supervision and administration. All authors contributed to the article and approved the submitted version.

## Funding

National Key Research and Development Program of China (2021YFC2301503), National Natural and Science Foundation of China (82171815, 82171739, 81873884), and Shanghai Science and Technology Commission (20Y11903400).

## Conflict of interest

The authors declare that the research was conducted in the absence of any commercial or financial relationships that could be construed as a potential conflict of interest.

## Publisher’s note

All claims expressed in this article are solely those of the authors and do not necessarily represent those of their affiliated organizations, or those of the publisher, the editors and the reviewers. Any product that may be evaluated in this article, or claim that may be made by its manufacturer, is not guaranteed or endorsed by the publisher.
