# Supplementary material for: Heterologous prime-boost BCG with DNA vaccine expressing fusion antigens Rv2299c and Ag85A improves protective efficacy against Mycobacterium tuberculosis in mice
Source: Front Microbiol. 2022 Oct 4;13:927031. doi: 10.3389/fmicb.2022.927031 (PMC9577005; doi:10.3389/fmicb.2022.927031)
Supplement: Supplementary file 1 [file Data_Sheet_1.docx]

**Heterologous Prime-Boost BCG with DNA Vaccines Expressing Fusion Antigens Rv2299c and Ag85A Improves Protective Efficacy against *Mycobacterium tuberculosis* in Mice**

Juan Wu^1^, Zhi-dong Hu^1^, Douglas B. Lowrie^2^, Shui-Hua Lu^2^, Xiao-Yong Fan^1*^

**Supplementary Figures**


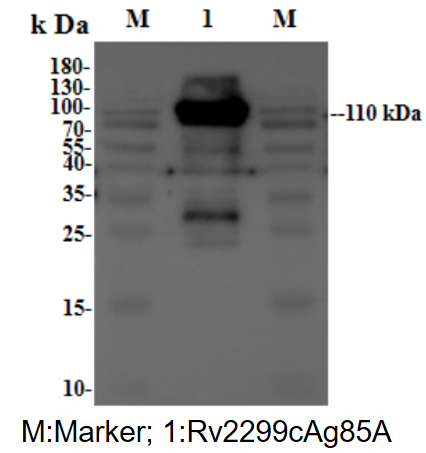


**Figure S1. Western blot demonstrating Rv2299cAg85A expression from the DNA fusion vaccine.** Lane M, Marker; Lane 1, lysate of 293T cells transfected with pVAX1 Rv2299cAg85A.


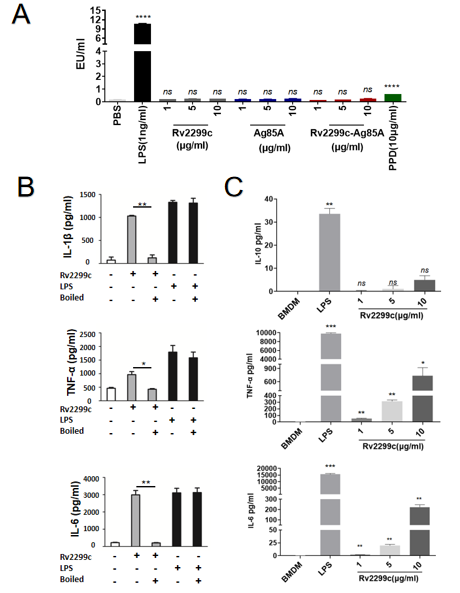


**Figure S2. Confirmation of endotoxin decontamination of the purified protein.** (A).Endotoxin content was measured by a LAL assay. (B). BMDCs (10^6^/well) were stimulated with LPS (100 ng/mL) or Rv2299c (10 μg/mL) for 24 h, the culture supernatant was determined by ELISAs. (C). Immature BMDMs (10^6^ cells/mL) were cultured in the presence of 1, 5, or 10 μg/mL Rv2299c or 100 ng/mL LPS for 24 h. The quantities of TNF-α,IL-6 and IL-10 in the culture supernatant were determined by ELISAs. (n=4-6,one-way ANOVA,mean±SEM ). *p < 0.05, **p < 0.01,***p < 0.001, ****p < 0.0001 : a significant difference of treatment groups from the appropriate controls(BMDM). *ns*: no significant difference.


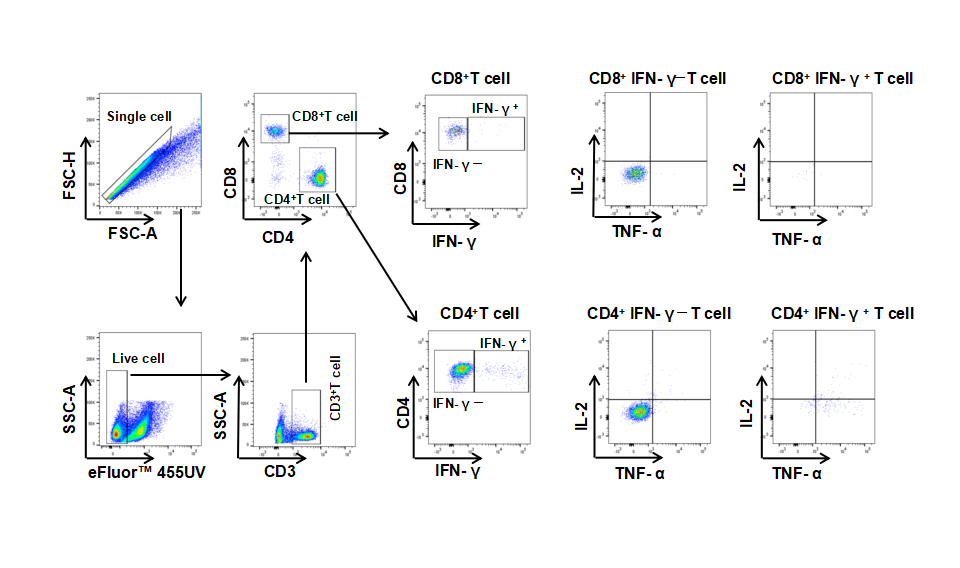


**Figure S3. Representative graphs showing the gating strategy for analysis of multi-cytokine-producing T cells.**


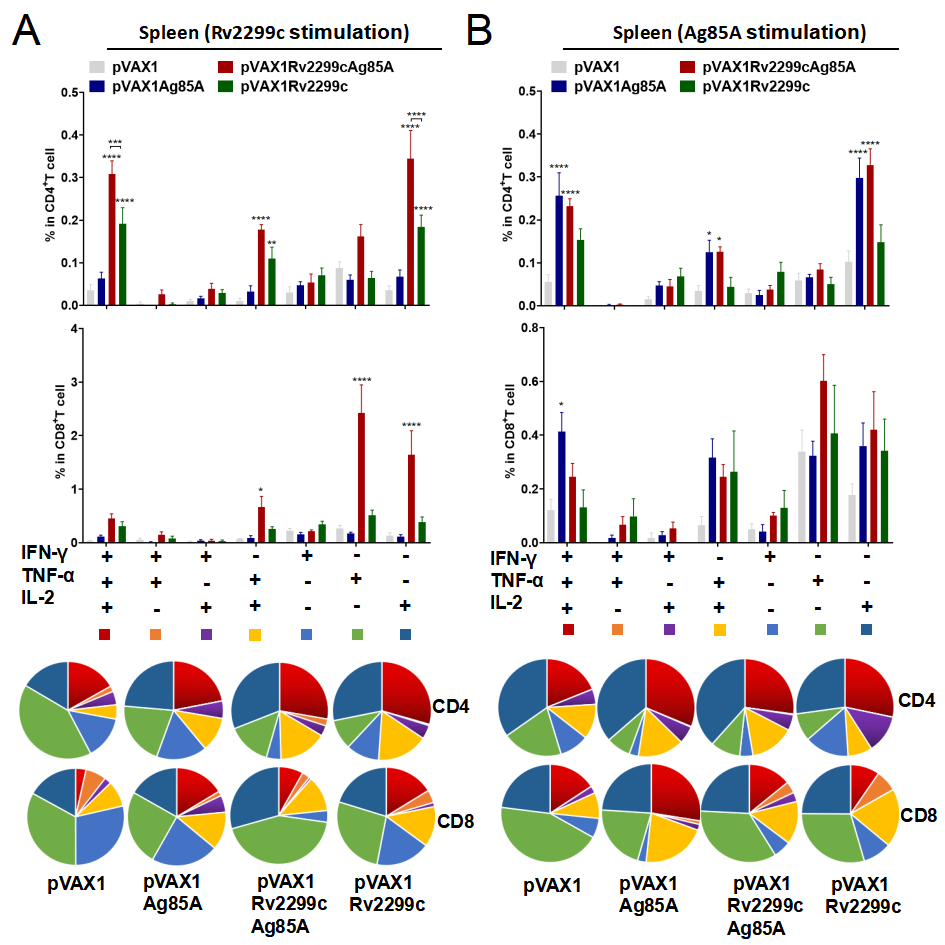


**Figure S4. Induction of Ag-specific polyfunctional T-cells in splenocytes of immunized mice.** Six weeks after the primary immunization, the mice were sacrificed, and splenocytes from different groups were collected and stimulated ex vivo with Rv2299c (A) or Ag85A (B). Frequencies of antigen-specific CD4^+^ (top) or CD8^+^ (center) T cells producing single or multiple cytokines were analyzed by flow cytometry. The pie chart analysis (bottom) is shown (*n* = 3 mice, two-way ANOVA, mean ± SEM).*p < 0.05, **p < 0.01,***p < 0.001, ****p < 0.0001 : a significant difference of treatment groups from the appropriate controls( pVAX1 group). *ns*: no significant difference.


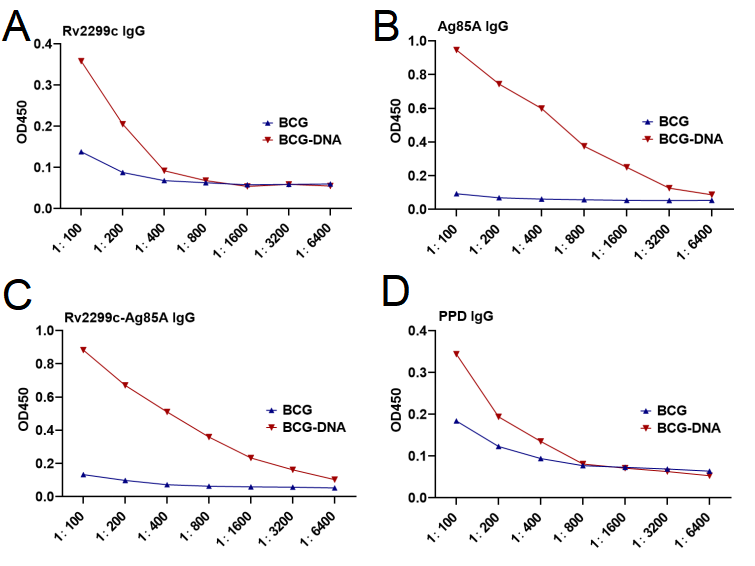


**Figure S5. Specific IgG antibody responses.** Six weeks after the primary immunization, the mice were sacrificed, serum was analyzed by ELISAs. (A,B,C,D). Specific IgG responses to antigen Rv2299c or Ag85A or Rv2299c-Ag85A or PPD in serum assayed six weeks after the primary immunization (n = 3 mice).


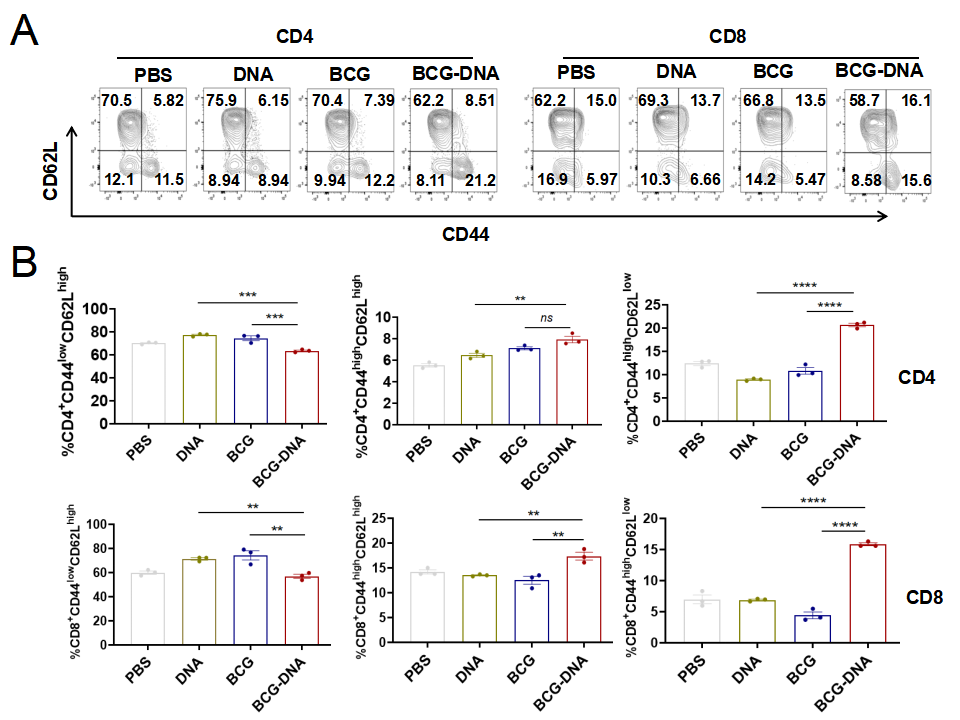


**Figure S6. BCG-primed DNA induce expansion of memory T-cell population.** Six weeks after the primary immunization, the mice were sacrificed, cells were harvested from the mice (n=3).Splenocytes were stained with anti-CD4, anti-CD8, anti-CD62L, and anti-CD44 monoclonal antibodies. (A). A histogram is shown for gating of the labeled T-cells. (B).Bar graphs show CD44^low^CD62L^high^ T-cells or CD44^high^CD62L^high^ T-cellsor CD44^high^CD62L^low^ T-cells populations among Splenocytes. (n = 3 mice, one-way ANOVA, mean ± SEM).*p < 0.05, **p < 0.01,***p < 0.001, ****p < 0.0001.


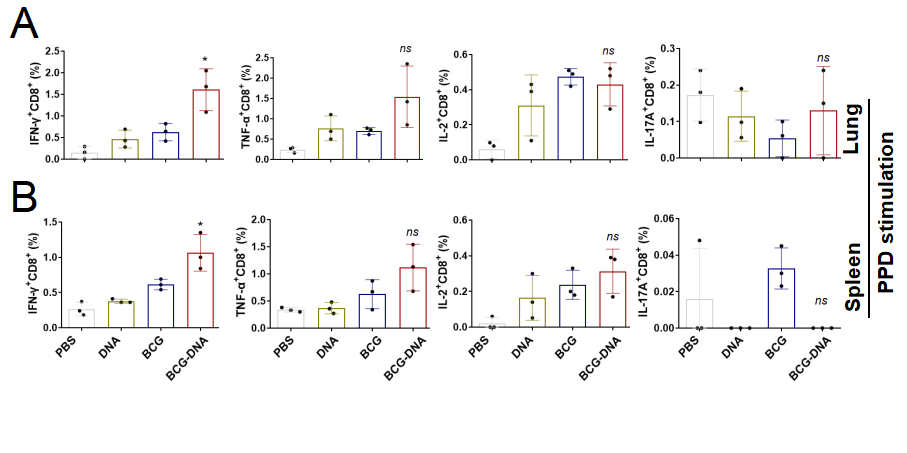
**Figure S7. Flow cytometric analysis of cytokines production in lung cells and splenocytes of immunized mice.** Six weeks after the primary immunization, the mice were sacrificed, and their lung cells and splenocytes collected from the mice (n=3) were treated with PPD (10 µg/ml) at 37℃ for 15 h in the presence of GolgiStop. Upon stimulation with PPD, Ag-specific CD8^+^ T cells producing IFN-γ, TNF-α, IL-2 and IL-17A in the lung cells (A) and splenocytes (B) from each immunized group were determined by flow cytometry and the proportion of cells producing these cytokines in CD8^+^ T cells are shown as histograms. (*n* = 3 mice, one-way ANOVA, mean ± SEM).*p < 0.05, **p < 0.01,***p < 0.001, ****p < 0.0001 : a significant difference of treatment groups from the appropriate controls( PBS group). *ns*: no significant difference.


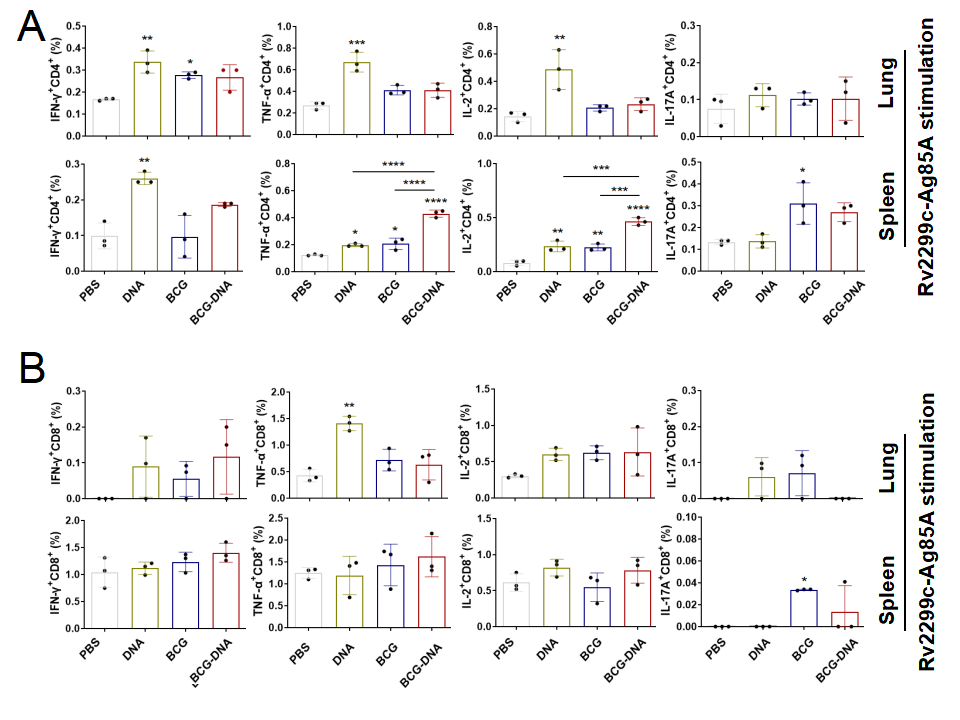


**Figure S8. Flow cytometric analysis of cytokine production in lung cells and splenocytes of immunized mice.** Six weeks after the primary immunization, mice were sacrificed, and their lung cells and splenocytes collected and treated with Rv2299c-Ag85A (10 µg/ml) at 37℃ for 15 h in the presence of GolgiStop. After stimulation with Rv2299c-Ag85A, Ag-specific CD4^+^ T cells and CD8^+^ T cells producing IFN-γ, TNF-α, IL-2 and IL-17A were determined by flow cytometry. The proportion of cells producing these cytokines in CD4^+^ T cells (A) and CD8^+^ T cells (B) are shown as histograms. (*n* = 3 mice, one-way ANOVA, mean ± SEM).*p < 0.05, **p < 0.01,***p < 0.001, ****p < 0.0001 : a significant difference of treatment groups from the appropriate controls( PBS group). *ns*: no significant difference.


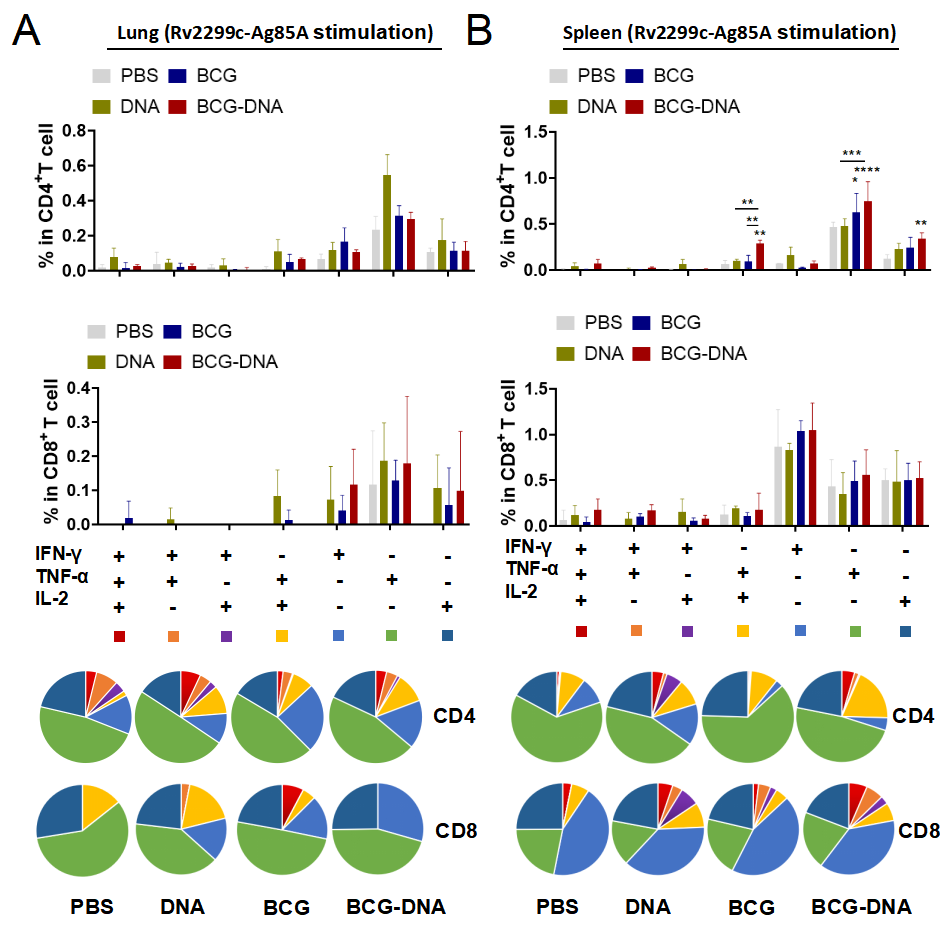
**Figure S9. Induction of Ag-specific polyfunctional T-cells in lung cells and splenocytes of immunized mice.** Six weeks after the primary immunization, the mice were sacrificed, and their lung cells and splenocytes collected and treated with Rv2299c-Ag85A (10 µg/ml) at 37℃ for 15 h in the presence of GolgiStop. After stimulation with Rv2299c-Ag85A, Ag-specific T cells secreting IFN-γ, TNF-α and IL-2 were distinguished as seven sub-population based on the secretion of these three intracellular cytokines in any combination. The percentage of the seven sub-populations as components of the total CD4^+^ (A top and B top) or CD8^+^ (A center and B center) T cells and the pie chart analysis (A bottom and B bottom) are shown. (*n* = 3 mice, two-way ANOVA, mean ± SEM).*p < 0.05, **p < 0.01,***p < 0.001, ****p < 0.0001 : a significant difference of treatment groups from the appropriate controls( PBS group). *ns*: no significant difference.
